# Enzymatic Production of Biologically Active 3-Methoxycinnamoylated Lysophosphatidylcholine via Regioselctive Lipase-Catalyzed Acidolysis

**DOI:** 10.3390/foods11010007

**Published:** 2021-12-21

**Authors:** Marta Okulus, Magdalena Rychlicka, Anna Gliszczyńska

**Affiliations:** Department of Chemistry, Wrocław University of Environmental and Life Sciences, Norwida 25, 50-375 Wrocław, Poland; marta.b.czarnecka@gmail.com (M.O.); rychlicka.magda@wp.pl (M.R.)

**Keywords:** acidolysis, lipases, Novozym 435, phosphatidylcholine, structured lipids, phospholipids, 3-methoxycinnamic acid

## Abstract

Enzymatic acidolysis of egg-yolk phosphatidylcholine (PC) with 3-methoxycinnamic acid (3-OMe-CA) was investigated to produce biologically active 3-methoxycinnamoylated phospholipids. Four commercially available lipases were screened for their ability to incorporate 3-OMe-CA into PC. The results showed that Novozym 435 is the most effective biocatalyst for this process, while during the examination of organic solvents, heptane was found propriate reaction medium. The other reaction parameters including the substrate molar ratio, enzyme load and reaction time were designed using an experimental factorial design method. According to three-level-3-factor Box-Behnken model it was shown that all of studied parameters are crucial variables for the maximization of the synthesis of structured PLs. The optimum conditions derived via response surface methodology (RSM) were: 30% of lipase of the total weight of substrates, 1:15 molar ration of PC/3-OMe-CA and reaction time 4 days. The process of acidolysis performed on the increased scale at optimized parameters afforded two products. The major product, 3-methoxycinnamoylated lysophosphatidylcholine (3-OMe-CA-LPC) was isolated in high 48% yield, while 3-methoxycinnamoylated phosphatidylcholine (3-OMe-CA-PC) was produced in trace amount only in 1.2% yield. Obtained results indicate that presented biotechnological method of synthesis of 3-methoxycinnamoylated lysophosphatidylcholine is competitive to the previously reported chemical one.

## 1. Introduction

The beneficial role of structured phospholipids (SPLs) in nutrition, health and food applications is enormous and many new literature reports in this area determine the constant increase of interest in this group of products and methods of their obtaining [1]. Compared to the chemical methods enzymatic modifications of phospholipids (PLs) are of special attention and are usually perform to alter and to improve the physiochemical or nutritional properties of PLs [1,2,3]. Their main advantage is selectivity of used for phospholipid modifications biocatalysts, which make the processes of production of SPLs simpler and let to conduct them under mild conditions [4,5]. The most popular biocatalysts mostly selected due to economic aspects are immobilized lipases. Their numerous advantages such as broad substrate specificity, ability to catalyze a large number of various reactions and no need to use of cofactors during reactions performed with their participation are responsible that they have become the object of special interest in food industry [6,7]. Their usage in food technology is also favored by European Union law, according to which products obtained as a result of biotransformation reactions of natural compounds are also classified as natural compounds (EU Directive 88/388/EEC). Therefore, the products of this type of reactions can be applied as health-promoting substances in the form of food additives or nutraceuticals.

Lipases can be divided into three groups taking into account their coordination-substrate site. First group are lipases from *Pseudomonas* and *Candida antarctica* family, which have active sites and funnel-like lids. *Candida antarctica* lipase B has a very small lid and a funnel-like binding site. Second group are lipases belonging to the *Rhizomucor* family including *Thermomycetes lanuginose*, which possess active sites and lids on the surface of the enzymes. The third group are those that have active sites at the ends of the tunnels containing the lids in their outer segments. This group includes lipases from the *Candida rugosa* family [8,9].

Phospholipids structured with phenolic acids and its *O*-methylated derivatives are presented in the literature as a novel group of biomolecules which can be dedicated for potential treatment of metabolic diseases, improvement body function and reduce risk of certain diseases. It was demonstrated that lysophosphatidylcholines conjugated with natural methoxy derivatives of benzoic acid may act as new insulin secretion modulators for pancreatic β-cells [10], whereas phosphatidylcholines with cinnamic acid and 3-methoxycinnamic acid have pro-health beneficial effect in restoring proper insulin sensitivity in insulin-resistant adipocytes [11]. Activity of phenolipids and its *O*-methylated derivatives towards selected cancer cell lines have been also evaluated and it has been indicated that these biomolecules are very promising anticancer agents [12,13,14].

Conjugation of phenolic acids with PLs increases their bioavailability and changes physiochemical properties affords not only the molecules with therapeutic potential but also new group of biosurfactants and antioxidants [15,16,17]. Therefore, lipids structured with phenolics are of great interest to both the pharmaceutical and cosmetic industries, as well as food industry [18,19].

**Scheme 1:**
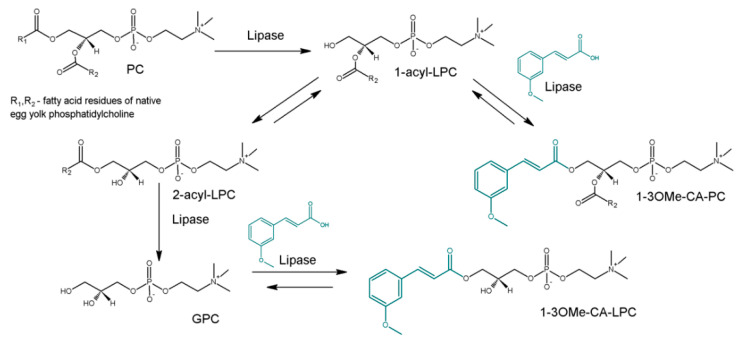
Lipase-catalyzed acidolysis of phosphatidylcholine with 3-methoxycinnamic acid.

The objective of our study was to develop and optimize the conditions of enzymatic production of 3-methoxycinnamoylated lysophosphatidylcholine for which it was proven that effectively inhibits the proliferation of leukemia cells at dose 30.5 μM being 11-fold more active than free 3-OMe-CA acid and 5-fold less toxic towards normal cells [13] as well as 3-methoxycinnamoylated phosphatidylcholine with antidiabetic properties [11]. Among the types of enzymatic reactions in which PL structured with 3-OMe-CA could be synthesized, we decided to investigate one-step enzymatic acidolysis. This type of reaction was chosen as a simple method, because during acidolysis process, the hydrolysis and esterification reactions take place in one step in one reaction vial at the same time. We have started experiments with selection of the most effective enzyme and organic solvent. Three next reaction parameters: substrate molar ratio, enzyme load and reaction time and their effects on the incorporation of 3-OMe-CA into phospholipid fraction were evaluated by using response surface methodology (RSM). We examined the relationship between reaction variables and the response (mole incorporation) using Box-Behnken experimental model. The yield of enzymatic synthesis of structured phospholipids was next determined with experiment conducted on the increased scale under the best conditions identified by RSM approach. Based on the obtained results we proposed the possible pathway of enzymatic acidolysis of PC with 3-methoxycinnamic acid (Scheme 1). Modification starts with hydrolysis at *sn*-1 position of phosphatidylcholine and successive esterification of this position with aromatic acid molecule leading to formation of the first reaction product modified phosphatidylcholine (1-3-OMe-CA-PC). However, simultaneously with the esterification process there occurs also a spontaneous migration of fatty acids from *sn*-2 to *sn*-1 position and formed LPC subsequently undergoes hydrolysis to *sn*-glycerophosphocholine (GPC). Further modification leads to formation of the second product of the reaction which is modified lysophosphatidylcholine (1-3-OMe-CA-LPC).

## 2. Materials and Methods

### 2.1. Reagents

Phosphatidylcholine (PC) used for acidolysis reaction was obtained from egg-yolk according to the previously described procedure [20].

Novozym^®^ 435 (>5000 U/gand CALB (>1800 U/g) were suplied by Sigma-Aldrich (St. Louis, MO, USA) while Lipozyme^®^ RM IM (>30 U/g) Lipozyme^®^ TL IM (250 U/g) were supplied by Fluka (Buchs, Switzerland) and Novozymes A/S (Bagsvaerd, Denmark), respectively. 3-Methoxycinnamic acid (3-OMe-CA), sodium methylate and boron trifluoride (13–15% BF_3_ in MeOH) were purchased from Sigma-Aldrich (St. Louis, MO, USA). Solvents used in the enzymatic reactions, silica gel (Kieselgel 60, 230–400 mesh) for column chromatography and silica gel-coated aluminum plates (Kieselgel 6-F254, 0.2 mm) were purchased from Merck (Darmstadt, Germany).

### 2.2. Acidolysis Reaction of Egg-Yolk Phosphatidylcholine with 3-Methoxycinnamic Acid

Research aimed at obtaining 3-methoxylated phospholipids began with the selection of an appropriate biocatalyst. Novozym 435, CALB, Lipozyme RM IM and Lipozyme TL IM were screened for acidolysis of phosphatidycholine (PC) with 3-methoxycinnamic acid (3-OMe-CA). Native phosphatidylcholine (20 mg, 0.026 mmol) was mixed with 3-OMe-CA (1/5 molar ratio) in 2 mL of heptane and N_2_ atmosphere, on the magnetic stirrer (300 rpm). Enzymatic modification was initiated when 30% of selected enzyme preparation (chosen as percentage of total weight of substrates) was added. Subsequently, the type of organic medium of reaction (toluene, isooctane, heptane) was also evaluated when Novozym 435 was used as a biocatalyst. All experiments were carried out in triplicates.

Acidolysis processes were terminated after 1–4 days. For this purpose, enzyme filtration (G4 Shott funnel) on Celite layer was applied. Next, phospholipid fractions were isolated from reaction mixture by silica gel columns and SPE methodology [20]. Formed phospholipid products were first confirmed by thin-layer chromatography (TLC) and next their fatty acids residues were quantitatively analyzed by gas chromatography (GC) procedure.

### 2.3. Design of Experiment

Box-Behnken design was utilized for optimization of acidolysis reaction parameters. The experimental design (3-factor and 3-level) included 15 trials (Table 1) with independent variables: substrate molar ratio of PC/3-OMe-CA (1/5, 1/10, 1/15), enzyme loading (10, 20, 30%) and reaction time (2, 3, 4 days) was used. The incorporation (mol%, based on GC) was the dependent variable. The polynomial equation of the model was as follows:

Y_i_ = β_0_ + β_1_X_1_ + β_2_X_2_ + β_3_X_3_ + β_12_X_1_X_2_ + β_13_X_1_X_3_ + β_23_X_2_X_3_ + β_11_X_1_^2^ + β_33_X_3_^2^

where Y_i_ is predicted response, β_0_ is model constant, X_1_–X_3_ are independent variables and β_1_—β_33_ are regression coefficient. The software used to interpret the obtained results was STATISTICA 13.3 (StatSoft, Inc.). All experiments were performed in duplicates.

### 2.4. Acidolysis of Egg-Yolk Phosphatidylcholine with 3-methoxycinnamic Acid Catalyzed by Novozym 435 in an Increased Scale

Acidolysis reaction was efficiently carried out in the large scale by mixing 200 mg (0.26 mmol) of egg-yolk phosphatidylcholine with 3-OMe-CA (molar ratio PC/3-OMe-CA, 1/15) in 20 mL of heptane. Enzymatic modification was performed on a magnetic stirrer (300 rpm) at 50 °C for 4 days in atmosphere of N_2_ and in a presence of 30% of Novozym 435. As before, the reaction was terminated by enzyme separation on Celite layer (G4 Shott funnel). In this case, phospholipid products were separated by column chromatography according to the procedure described before [21]. Purified products fractions: PC-egg, LPC-egg, modified phosphatidylcholine (3-OMe-CA-PC) and modified lysophosphatidylcholine (3-OMe-CA-LPC) were confirmed by thin layer chromatography (TLC), gas chromatography (GC) and high-performance liquid chromatography (HPLC).

### 2.5. Methods of Analysis

#### 2.5.1. Thin-Layer Chromatography (TLC)

Thin-layer chromatography (TLC) was applied as a quick method to control the progress of the reaction at each stage of the research. TLC were performed on 0.2 mm silica gel 60 F245 plates (Merck, Darmstadt, Germany). The eluent used for developing the chromatograms was a mixture of chloroform/methanol/water (65:25:4, *v*/*v*/*v*), while the 0.05% primuline solution in mixture of acetone:water, 8:2, *v*/*v*) was used for products identification under UV light (λ = 365 nm). R_f_ of different bands was identified as follows: 0.28 PC-egg, 0.12 LPC-egg, 0.21 3-OMe-CA-PC and 0.07 3-OMe-CA-LPC.

#### 2.5.2. Gas Chromatography (GC)

Gas chromatography (GC) was applied for analysis of acid profile of acidolysis products (PC-egg, LPC-egg, 3-OMe-CA-PC, 3-OMe-CA-LPC) and standards (PC-egg, 3-OMe-CA). For this purpose purified samples (SPE/column chromatography methods) were derivatized into corresponding to them methyl esters as described before [22] and analyzed on an Agilent 6890N [20]. Retention times of products were compared with retention time of a standard (Supelco 37 FAME Mix, Sigma Aldrich).

#### 2.5.3. High-Performance Liquid Chromatography (HPLC)

The composition of the mixture of products obtained as a result of the optimized acidolysis was also analyzed by HPLC on DIONEX UltiMate 3000 chromatograph Thermo Fisher Scientific (Olten, Switzerland) equipped with UV/CAD detector. Due to significant differences in lipophilic/hydrophilic character of products two different columns were applied. PC-egg, LPC-egg, 3-OMe-CA-PC were analyzed with BetaSil DIOL column (250 × 4.6 mm, 5 μm). In turn more hydrophilic 3-OMe-CA-LPC was confirmed with Ascentis express C18 column (150 × 4.6 mm, 5 μm). All separation parameters were set according to the procedure described earlier [21].

## 3. Results and Discussion

### 3.1. Screening of Commercial Immobilized Lipases

Lipases are commercially available from several suppliers under free or immobilized form. Moreover, the currently available preparations of lipases immobilized on a hydrophobic substrate enable the catalysis of the reaction in the presence of an organic solvent which are dedicated to lipid modifications and high concentrations of substrates while maintaining a satisfactory efficiency and selectivity of the process [23]. In our study we used lipases in immobilized form. Lipase from *Rhizomucor miehei* RM IM (Lipozyme) was immobilized on an anion exchange resin whereas lipase from *Theromomyces lanuginosus* was used in a silica granulated form. Preparations containing the same lipase B from *Candida antarctica* were differ of carriers. In the case of preparation of Novozym 435 lipase B was immobilized on a microporous acrylic resin while in the case of CALB the same enzyme was immobilized on resin Immobead 150.

All of mentioned preparations of lipases were evaluated to catalyzed acidolysis of natural phosphatidylcholine (PC) with 3-methoxycinnamic acid (3-OMe-CA). During the selection of biocatalysts, we focused our attention on the literature data and previously reported results from enzymatic modifications of phospholipids [17,22,24,25]. We selected the lipases from different groups and immobilized on different carriers, which are determined as significantly selective towards the *sn*-1 position of triacylglicerols and phospholipids such as Lipozyme RM IM and Lipozyme TL IM [26,27] or classified as non-specific such as Novozym 435 and CALB but shows in most studied cases of enzymatic modifications of lipids high selectivity towards the *sn*-1 position [28]. Then we focused on selection of a reaction temperature suitable for all these lipase preparations. We analyzed literature data and description of optimum usage conditions for lipases and we found that the reaction temperature will be set at 50 °C [29,30].

**Figure 1:**
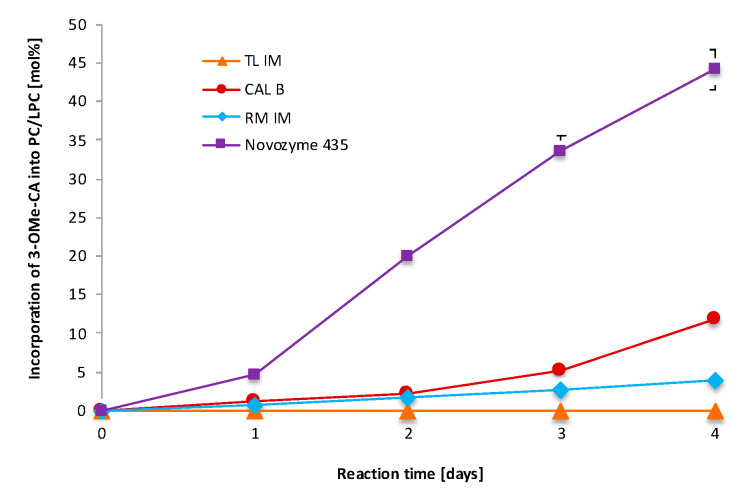
Dependence of incorporation degree of 3-OMe-CA into egg-yolk PC on reaction time for different lipases (reaction conditions: 1:5 PC/3-OMe-CA molar ratio, solvent: heptane 2 mL, enzyme load 30% (*w*/*w*), temperature 50 °C). Vertical bars represent SD, *n* = 3.

At this stage of experiments reactions were conducted at 50 °C in 2 mL of heptane. Substrates PC/3-OMe-CA used in the molar ratio 1:5 were mixed with 30% (*w*/*w*) of dosage of commercial lipase preparation. The samples of reaction mixtures were collected after 1, 2, 3 and 4 days and phospholipid fractions PC/LPC were purified from fatty acids and 3-methoxycinnamic acid by solid phase extraction (SPE). Next the samples after previous derivatization were analyzed by gas chromatography (GC). On Figure 1 the time course of incorporation of 3-OMe-CA into phospholipid fraction (PC/LPC) by studied enzyme preparations is shown. It is visible that 2-day reaction resulted in the highest incorporation of aromatic acid when the process was catalyzed by Novozym 435 and it was great higher than that reported for other tested lipases. Incorporation increased when reaction time was extended and reached 44 mol% after 4 days using Novozym 435 as a biocatalyst whereas acidolysis catalyzed with CALB resulted in lower incorporation degree—only 12 mol%. The Lipozyme RM IM showed the lowest activity to catalyze the incorporation of 3-OMe-CA into phospholipids (3.8 mol%) while Lipozyme TL IM turned out to be inactive in this reaction.

Based on the obtained results (Figure 1) we selected as the most effective biocatalyst able to catalyze the reaction of acidolysis of phosphatidylcholine and 3-OMe-CA Novozym 435 which was also the most efficient in previously studied synthesis of phenophospholipids [21,22,24,25]. Our results somehow confirm why Novozym 435 is the most used lipase preparation in the literature. We present that this preparation can be successfully employed also as biocatalyst in the enzymatic production of phospholipids structured with aromatic acids almost regardless of their chemical structure, so their substrate specificity is enormous. Analyzing the reported in recent years results from enzymatic modifications of fatty alcohols, TAGs and PLs with phenolic acids it looks that the steric hindrance which make unable to incorporate the phenolic acids occur when two methoxy or two hydroxy groups are substituted at the benzene ring next to each other [25,31,32,33] in other case Novozym is able to catalyze the reaction of exchange the acyl groups.

### 3.2. Effect of Organic Solvents on Enzyme Activity

**Figure 2:**
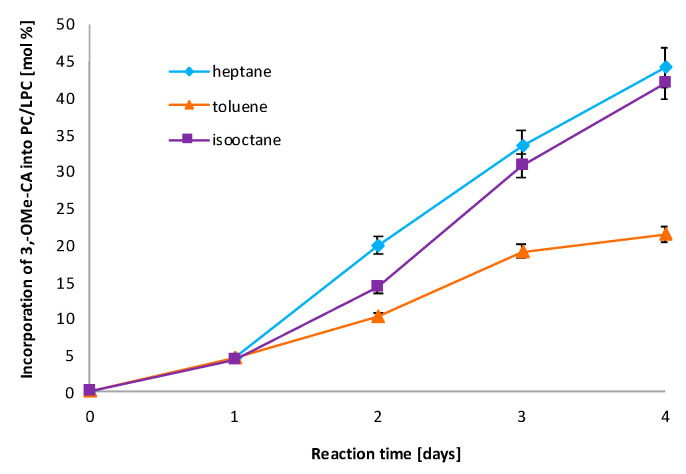
Dependence of incorporation degree of 3-OMe-CA into egg-yolk PC on reaction time for different organic medium (reaction conditions: Novozym 435, 1:5 PC/3-OMe-CA molar ratio, solvent 2 mL, enzyme load 30% (*w*/*w*), temperature 50 °C, reaction time 1, 2, 3 and 4 days). Vertical bars represent SD, *n* = 3.

The preparations of immobilized lipases allow for conducting the process of lipid modification in the anhydrous environment of an organic solvent, what has a significant impact on the efficiency of the enzymatic process [34]. During the enzymatic synthesis of modified PLs we also had to take into account that 3-methoxycinnamic acid, due to its hydrophilic character, is not a typical substrate for lipases and shows limited solubility in such media. We studied three different organic solvents in the next step of optimization process of the lipase-catalyzed synthesis of 3-methoxycinnamoylated phospholipids. Selected solvents were characterized by different polarity with log P (logarithum of partition coefficient of organic solvent in water and 1-octanol) ranging from 2.5 (toluene) through 4.0 (heptane) to 4.5 (isooctane). The results are shown in Figure 2. In our studies the highest incorporation 44 mol% was achieved in heptane however it was similar to corresponding incorporation when isooctane was used (41 mol%) as a reaction medium. During the reaction performed in toluene much lower degree of incorporation of 3-OMe-CA into phospholipid fraction was observed. Based on the obtained results it is clearly visible that better effects were achieved in non-polar solvents. This result agrees with literature where has been noted that solvents with logP > 4 are convenient for biocatalysis since they do not remove essential water layer around the enzyme [35]. Therefore, in solvents with low logP a decrease in product formation occurs. Higher incorporation in heptane than in more non-polar hexane is contrary to the principle mentioned above however is in accordance in previously observed tendency during enzymatic modifications of PC with anisic acid. In that case also higher level of incorporation was observed in the heptane than in isooctane. However, in that case due to low solubility of anisic acid better medium turned out to be the mixture of toluene and chloroform [25]. Therefore, the environment of the modification reaction seems to be a parameter having a significant influence on the process, which should be chosen individually for each substrate.

### 3.3. Model Fitting

In order to minimize costs and at the same time to better understand the relationship between the values of dependent and independent variables, the statistical method of designing the experiment was used in the research. Among the many available models of biocatalytic processes, the Box-Behnken (BBD) design was used as the one characterized by high efficiency [36]. A significant advantage of BBD is the ability to construct a second-order polynomial equation, and consequently also response surface plots useful for predicting the value of the dependent variable from differences in the values of the independent variables, which significantly reduces the number of trials necessary to draw conclusions about the course of the process under study.

In the next step of our studies, we employed the response surface methodology (RSM) which is more efficient method than changing one-factor-at-a-time, resulting in reduction of needed experimental trials (15 trials with 3 replicates at the central point) as well as time needed for optimization of enzymatic production of phosphatidylcholine structured with 3-methoxycinnamic acid. Box-Behnken experimental design with selected 3 independent variables substrate molar ration, enzyme loading and reaction time at 3 different levels was used to study the effects on dependent variable level of incorporation. The data given below in Table 1 show experimental and predicted values of incorporation of 3-OMe-CA into PLs fraction expressed in mol%. The predicted values were obtained from a model fitting technique using the software Statistica 13.3 (StatSoft, Inc.).

The analysis of variance (ANOVA) data is presented in Table 2. This model was found to have coefficient of determination value (R^2^) of 0.98771, which means that 98.771% of total variation in the observed findings was attributed to the independent variables. This indicates a good agreement between predicted and experimental values of degree of incorporation.

**Figure 3:**
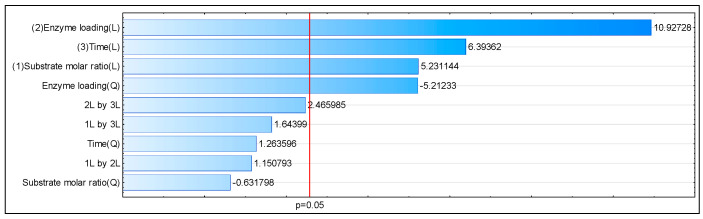
Pareto diagram for the effects of the analyzed variables.

In general, value of calculated model coefficients should be as high as possible in the case of *F*, and in the case of *p* less than 0.05 so that the analyzed variable has a statistically significant influence on the course of the process [37]. Thus, based on data presented on Pareto chart (Figure 3), we assumed that in our studies enzyme loading (L and Q), time (L) and substrate molar ration (L) have a significant effect on incorporation. It should also be noted that most of the bar values are positive, which means that as the bars increase, the incorporation rate is expected to increase. Only enzyme loading (Q) was characterized by a minus sign, which suggests that this parameter may have an inverse effect on the degree of incorporation and cause its decrease.

**Figure 4:**
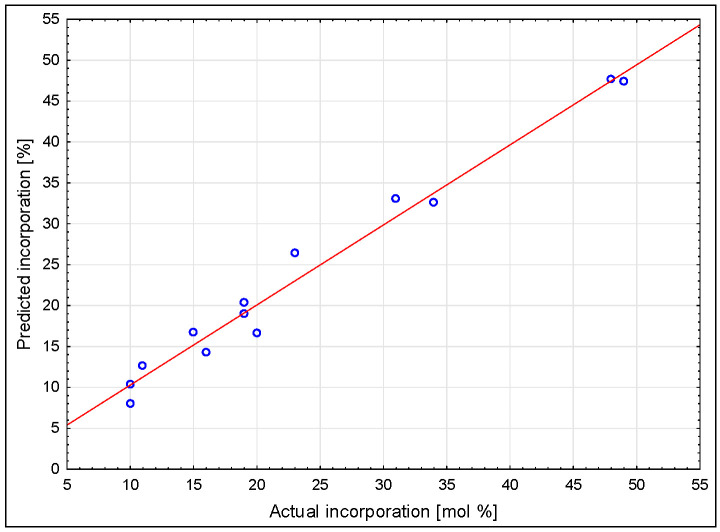
Comparison of predicted and actual incorporation of 3-OMe-CA into phospholipid fraction.

Figure 4 presents good correlation between the experimental (actual) and predicted incorporation degree for acidolysis of PC with 3-methoxycinnamic acid using Novozyme 435 and carried out the reaction in heptane. The linear distribution is indicative of a well-fitted model.

### 3.4. Response Surface Metodology Analysis and Interpretation

**Figure 5:**
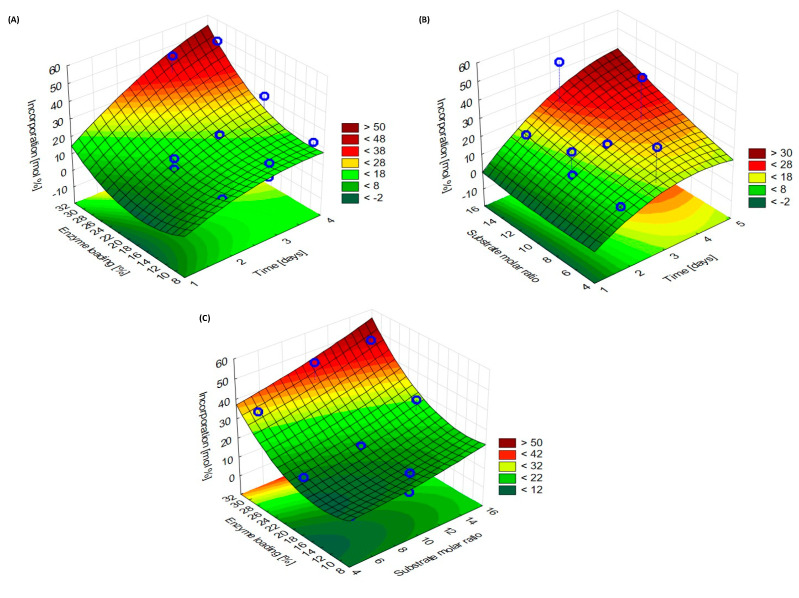
Response surface plots presenting effect of (**A**) enzyme loading and time of reaction, (**B**) substrate molar ratio and time of reaction, (**C**) enzyme loading and substrate molar ratio on the incorporation of 3-OMe-CA into phospholipid fraction.

Three-dimensional contour plots formed based on the model equation allowed us for visualization of the reaction system and led to understand the interactions between used variables. The effects of the studied factors on the degree of incorporation are presented on Figure 5A–C.

As shown in Figure 5A the process of incorporation of 3-OMe-CA into PC significantly depends on enzyme loading and time of the reaction and increases with increase of both of these parameters. The highest experimentally determined degree of incorporation of aromatic acid into phospholipid fraction was obtained by usage during the acidolysis reaction 30% (*w*/*w*) enzyme dosage and performs reaction by 3 days. The presence of higher amount of enzyme provides more active sites for acyl-enzyme complex formation and also increases the probability of enzyme-substrate collision and subsequent reaction [38]. However, it is important to remember that this tendency takes place only until some specific level after which the negative effect of further increase in enzyme concentration may be due to mass transfer limitation and poor dispersion of enzyme particles [39].

The influence of used quantity of substrates and reaction time on the degree of incorporation is shown in Figure 5B, when the amount of enzyme was selected as the center point. As can be seen, reaction with the highest substrate molar ratio 1:15 (PC/3-OMe-CA) and highest time favored maximal incorporation. We did not observe during the enzymatic synthesis PLs structured with 3-OMe-CA but this parameter has also its own limitation with problems with solubility of high amount of substrate. Above solubility limitation the viscosity increase and mass transfer decrease thus might result in a decrease of volumetric productivity.

Figure 5C shows the effect of enzyme loading and substrate molar ratio on the incorporation of aromatic acid into phosphatidylcholine. It is well known that both of these parameters are one of the most important factors. The response increases as the enzyme content and molar ratio are raised. When enzyme dose and substrate ratio was low, the incorporation degree was inferior. Acidolysis conducted with higher amount of these two parameters afforded more product. Presence of larger quantity of substrates generally increases the probability of substrate enzyme collision [40]. Similarly higher amount of enzyme increases percentage of incorporation, but these trends can be observed only until certain point, after which the problems with viscosity and mixing of reaction mixture always occurs. On the other hand, it was reported in the literature that satisfying conversion is achievable even when enzyme dose is low and amount of substrate is high. This approach is also relevant from the economic due to that cost of enzyme is usually higher than that of substrate [41,42].

Three-dimensional plots of the response surface allowed for a pragmatic and at the same time understandable analysis of the relationship between the input parameters and the obtained response value. Each of them illustrates the dependence of the incorporation on the influence of two different reaction variables, while the third independent is fixed at the central value. Generally, among possible types of plots of RSM for lipase catalyzed esterifications four can be distinguish [43]. In the case of studied acidolysis of PC with 3-OMe-CA type III was characteristic, where almost linear relation was observed and increase in extent of esterification with increase in both studied variables. Looking on the formed contour plot we could observe that it is possible to achieve even a little bit higher degree of synthesis of structured PC by prolonging the time of reaction by one more day. Then maximum theoretical incorporation 50 mol% possible to obtain during enzymatic modifications performs with usage of Novozym 435 which regioselectively catalyzed the exchange of acyl fragments mostly at position *sn*-1. Keeping in mind that enzyme loading in quadratic equation had a negative influence what was presented on Pareto chart we concluded that there is no point to increase more dosage of used lipase preparation and only increase of time is justified.

To further validate the accuracy of RSM prediction an experiment was performed using the predicted optimal composition. Under the parameters set as follows: reaction medium heptane, 1:15 PC/3-OMe-CA molar ratio, Novozym 435 (30% *w*/*w* dosage) and time 4 days we carried out the reaction of acidolysis of natural phosphatidylcholine with 3-methoxycinnamic acid on the increased scale. Formed products were identified based on the available standards of chemically obtained structured PC and LPC which were previously reported (Supplementary Materials: Figure S1, Figure S2). Analysis of the acids’ composition of modified phospholipid fraction (PC/LPC) indicated the high content of 3-methoxycinnamic acid 58 mol% and small amounts of other acids (about 10 mol% C16:0 and 3 mol% C18:0) (Table 3). The increase of aromatic acid was accompanied by a reduction of saturated fatty acids, which occur in the *sn*-1 position of phosphatidylcholine isolated from egg-yolk (Supplementary Materials: Figure S3).

Based on TLC and HPLC results we could establish that the main product of the reaction 3-methoxycinnamoylated-LPC (3-OMe-CA-LPC) was obtained in high 48% of isolated yield whereas second product 3-methoxycinnamoylated-PC (3-OMe-CA-PC) was obtained in trace amount only in 1.2% isolated yield.

## 4. Conclusions

The study showed that Novozym 435 (immobilized lipase B from *Candida Antarctica*) is a good catalyst choice for synthesis of structured phospholipids in the acidolysis of phosphatidylcholine with 3-methoxycinnamic acid in organic medium. Using this enzyme, it was able to catalyze the incorporation of 3-OMe-CA into an apolar part of PC in the *sn*-1 position where saturated fatty acids naturally occur. The analysis of the effects of selected reaction parameters on diverse response variables showed that enzyme loading, time and substrate molar ration are key factors for modulating the incorporation of 3-OMe-CA into phospholipid fraction. Under the optimized parameters the best incorporation of 3-OMe-CA into phospholipid fraction reached 58 mol%. 3-Methoxycinnamoylated-LPC (3-OMe-CA-LPC) and 3-methoxycinnamoylated-PC (3-OMe-CA-PC) were obtained in isolated yields 48% and 1.2% (*w*/*w*), respectively.

In order to compare the costs of obtaining 3-OMe-CA-LPC in laboratory conditions, we compared the costs of purchasing substrates and reagents required for carry out the developed chemical and enzymatic methods. Per one reaction and not taking into account the purchase of PC, which we have isolated from natural sources, the cost of producing 3-OMe-CA-LPC by chemical synthesis is about 11 times lower. Nevertheless, considering that the immobilized enzyme preparation can be used even up to 10 times in the synthesis, what was tested in laboratory conditions, the difference in the production price is significantly lower. Then the cost of obtaining modified lysophosphatidylcholine by enzymatic synthesis is only 3 times higher, and what is crucial the proposed method is good and an environmentally friendly alternative. Therefore, presented method is promising in the area of enzymatic production of phospholipid biopreparation containing biologically active molecules with potential application as food additives or nutraceuticals with pro-health activity and great alternative to previously described chemical synthesis.

## Figures and Tables

**Table 1:** Experimental design matrix for three independent variables of enzymatic acidolysis of egg-yolk phosphatidylcholine (PC) with 3-methoxycinnamic acid (3-OMe-CA).

Run	Substrate Molar Ratio PC/3-OMe-CA	Enzyme Loading [%]	Reaction Time [Days]	Incorporation of 3-OMe-CA into PC/LPC [mol%] ^a^ (Experimental)	Incorporation of 3-OMe-CA to PC/LPC [mol%] (Predicted)
1	5	10	3	11 ± 0.4	13
2	15	10	3	19 ± 0.6	20
3	5	30	3	34 ± 1.2	33
4	15	30	3	49 ± 2.3	47
5	5	20	2	10 ± 0.6	8
6	15	20	2	16 ± 0.9	14
7	5	20	4	15 ± 0.7	17
8	15	20	4	31 ± 1.1	33
9	10	10	2	10 ± 0.5	10
10	10	30	2	23 ± 0.9	26
11	10	10	4	20 ± 0.7	17
12	10	30	4	48 ± 1.8	48
13	10	20	3	19 ± 0.7	19
14	10	20	3	19 ± 0.6	19
15	10	20	3	19 ± 0.2	19

^a^ Data are presented as mean ± standard deviation (SD) of two independent analyses.

**Table 2:** Analysis of variance (ANOVA) for acidolysis variables pertaining to the response of percent incorporation of 3-OMe-CA into PC/LPC.

Evaluated Factors	Sum of Squares	Degrees of Freedom	Medium Square	F-Value	*p*-Value
(1) Substrate molar ratio (L)	253.1250	1	253.125	27.3649	0.003379
Substrate molar ratio (Q)	3.692	1	3.692	0.3992	0.555275
(2) Enzyme loading(L)	1104.500	1	1104.500	119.4054	0.000112
Enzyme loading (Q)	251.308	1	251.308	27.1684	0.003432
(3) Time of reaction (L)	378.125	1	378.125	40.8784	0.001387
Time of reaction (Q)	14.769	1	14.769	1.5967	0.262086
1 by 2	12.250	1	12.250	1.3243	0.301861
1 by 3	25.000	1	25.000	2.7027	0.161099
2 by 3	56.250	1	56.250	6.0811	0.056810
Error	46.250	5	9.250		
Total error	2153.733	14			
R^2^ = 0.98771					

**Table 3:** Composition of fatty acids (mol%) of native egg-yolk PC and modified PC.

**Fatty and 3-OMe-CA Acids**	**Native PC**	**Modified PC**
C16:0 (PA)	36	10
C16:1 (OPA)	3	1
C18:0 (SA)	16	3
C18:1 (OA)	23	13
C18:2 (LA)	17	12
C20:4 (AA)	5	3
3-OMe-CA	-	58

## Data Availability

Data are contained within the article.

## Supplementary Materials

The following are available online at https://www.mdpi.com/article/10.3390/foods11010007/s1. Figure S1: HPLC chromatogram of 3-OMe-CA-PC; Figure S2: HPLC chromatogram of 3-OMe-CA-LPC; Figure S3: GC chromatogram of fatty acid composition of modified phospholipid fraction PC/LPC.
