# Supplementary material for: Enzymatic Production of Biologically Active 3-Methoxycinnamoylated Lysophosphatidylcholine via Regioselctive Lipase-Catalyzed Acidolysis
Source: Foods. 2021 Dec 21;11(1):7. doi: 10.3390/foods11010007 (PMC8750904; doi:10.3390/foods11010007)

Supplementary Materials

Enzymatic Production of Biologically Active 3-Methoxycinnamoylated Lysophosphatidylcholine via Regioselctive Lipase-catalyzed Acidolysis

Marta Okulus, Magdalena Rychlicka and Anna Gliszczyńska *

Department of Chemistry, Wrocław University of Environmental and Life Sciences, Norwida 25, 50-375 Wrocław, Poland; marta.b.czarnecka@gmail.com (M.O.); rychlicka.magda@wp.pl (M.R.)

* Correspondence: anna.gliszczynska@wp.pl (A.G.); Tel.: +48-71-320-5183

Received: 9 November 2021; Accepted: 16 December 2021; Published: 21 December 2021

Content

Figure S1: HPLC chromatogram of 3-OMe-CA-PC. 2

Figure S2: HPLC chromatogram of 3-OMe-CA-LPC. 3

Figure S3: GC chromatogram of fatty acid composition of modified phospholipid fraction PC/LPC. 4

Figure S1: HPLC chromatogram of 3-OMe-CA-PC.

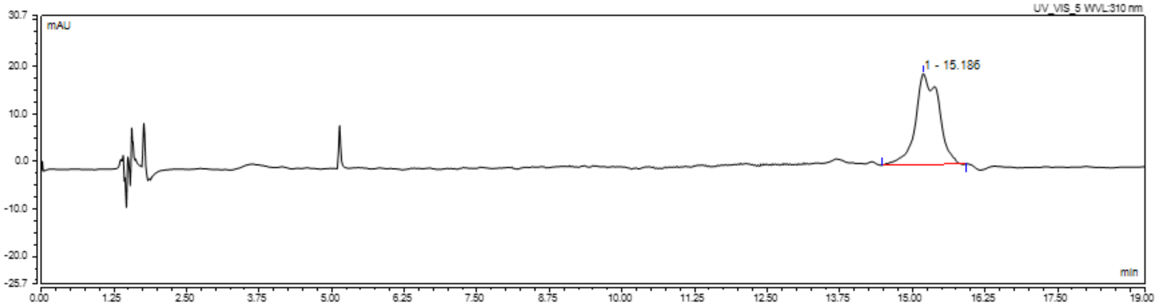

Figure S2: HPLC chromatogram of 3-OMe-CA-LPC.

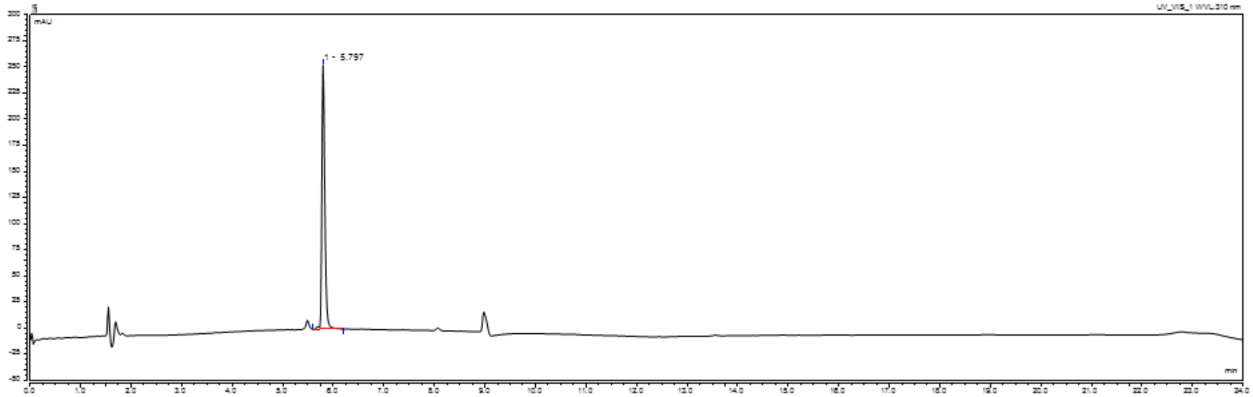

Figure S3: GC chromatogram of fatty acid composition of modified phospholipid fraction PC/LPC.

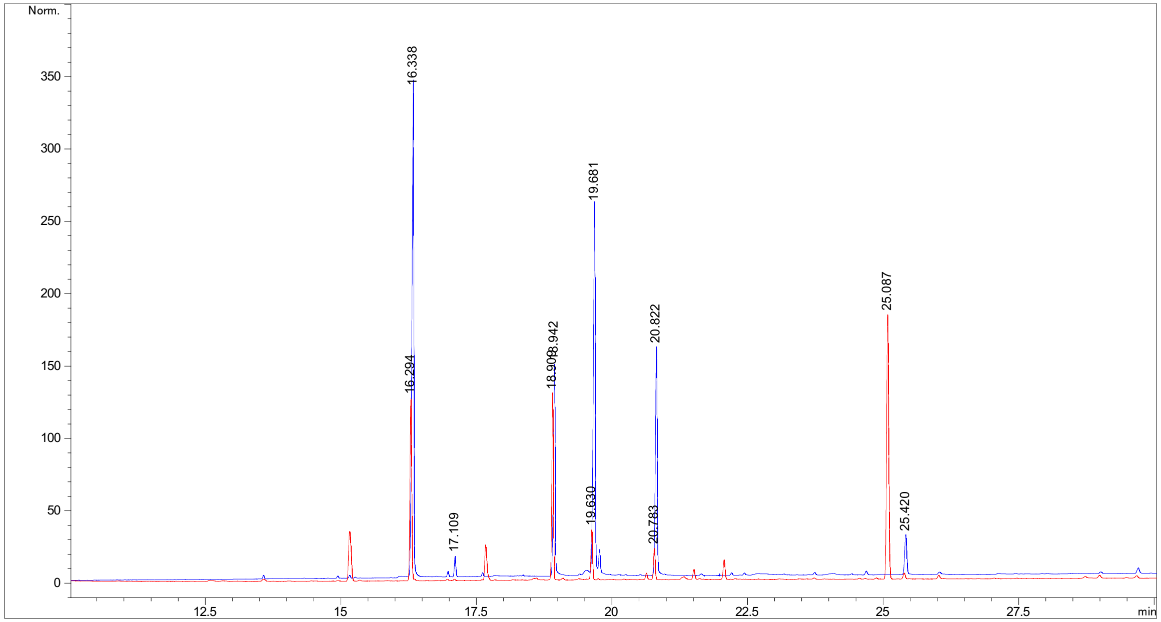

Supplement: Supplementary file 1 [file foods-11-00007-s001.zip › foods-1479587-SI.pdf]
